# Accurate Fall Detection in a Top View Privacy Preserving Configuration

**DOI:** 10.3390/s18061754

**Published:** 2018-05-29

**Authors:** Manola Ricciuti, Susanna Spinsante, Ennio Gambi

**Affiliations:** Dipartimento di Ingegneria dell’Informazione, Università Politecnica delle Marche via Brecce Bianche 12, 60131 Ancona, Italy; s.spinsante@univpm.it (S.S.); e.gambi@univpm.it (E.G.)

**Keywords:** fall detection, ADLs, Kinect, depth frame, privacy, machine learning, elderly people

## Abstract

Fall detection is one of the most investigated themes in the research on assistive solutions for aged people. In particular, a false-alarm-free discrimination between falls and non-falls is indispensable, especially to assist elderly people living alone. Current technological solutions designed to monitor several types of activities in indoor environments can guarantee absolute privacy to the people that decide to rely on them. Devices integrating RGB and depth cameras, such as the Microsoft Kinect, can ensure privacy and anonymity, since the depth information is considered to extract only meaningful information from video streams. In this paper, we propose an accurate fall detection method investigating the depth frames of the human body using a single device in a top-view configuration, with the subjects located under the device inside a room. Features extracted from depth frames train a classifier based on a binary support vector machine learning algorithm. The dataset includes 32 falls and 8 activities considered for comparison, for a total of 800 sequences performed by 20 adults. The system showed an accuracy of 98.6% and only one false positive.

## 1. Introduction

In contemporary society, the aging of the population is a growing phenomenon that requires investments [1] for the development of assisted living environments which are able to provide technological tools to support active aging in nursing homes or in health care facilities. Among the Ambient Assisted Living (AAL) research and development issues, the availability of automatic fall detection solutions could reduce the risk of complications deriving from a critical event [2] (e.g., femoral fracture) caused by a fall. The study presented herein stems from the awareness that real-time near-fall detection is a very important factor, because it could prevent fall risk and even death, especially for elderly people.

The aim of this work is therefore to design an automatic system for fall detection based on the depth data extracted from the Kinect sensor. The choice of this technology was motivated by the possibility of realizing an unobtrusive monitoring of the environment of interest, guaranteeing respect for user privacy. By evaluating the use of several currently available technologies, it is possible to consider the following issues and typologies:

- *Wearable sensors*: usually represented by accelerometers [3] positioned on human body parts, equipped with a battery and a wireless communication interface. The use of this technology requires the collaboration of the subjects wearing the device and recharging the battery, with the assumption that subjects affected by neurological problems could find it difficult to carry out these procedures.
- *Ambient sensors*: magnetic sensors for doors and windows, or bed and armchair sensors, which provide information on the interaction of the monitored subject with the objects. In contrast to the wearable sensors, this technology does not require subject collaboration, but the provided information is used for some specific application [4,5,6].
- *Video cameras*: the classic technology [7] for monitoring domestic and non-domestic environments. The use of this type of sensor for fall detection could generate problems in some situations. Firstly, because the video captured by the cameras depends on environmental lighting, sometimes strong variations in brightness make it difficult to capture images or videos of sufficient quality. In addition, the installation of the cameras in rooms such as bathrooms or bedrooms can generate problems with privacy preservation. The advantage of the cameras is to have a wider field of view than the depth sensors, but this drawback could be solved by installing multiple depth devices to cover a larger area. Moreover, depth sensors [8,9] allow privacy preservation, since it is not possible to recognize faces or other personal details from depth images.

The work presented herein takes its foundation from the functionalities of an algorithm for fall detection [10] based on depth frames extracted from the Kinect sensor in a top view configuration. This fall detection algorithm [10] was based on the Kinect v1 sensor, installed on the ceiling at 3 m from the floor, with the aim of monitoring falls in a domestic environment. By processing only the depth frame and using data characterizing human subjects with geometric features and anthropometric relationships, this algorithm was able to detect the different objects in the scene, identify the presence of a person, and monitor the person’s conditions by evaluating the distance between the subject and the floor. The main limit of this system was the ability to recognize only falls in which the person was lying on the ground. On the contrary, other types of falls were not detected, especially when the person fell in a sitting position or on the knees. The first phase of the proposed work was therefore to include a number of activities of daily living (ADLs) in the fall data acquisition protocol, performed by a considerable number of subjects, to identify the situations in which the previous algorithm was not able to distinguish falls in order to modify it and improve its performance. In contrast to most of the literature on fall detection, which use the sensor in a front view configuration, in this project we use Kinect in a top-down view. The sensor was installed on the ceiling of a room (or rather the laboratory where tests were performed). The Kinect device is able to calculate the subject’s skeleton and the joints’ coordinates when used in front view, but in this case where the view is from top to bottom, the skeleton is not available for processing. In this paper, the fall detection capability takes place on depth frames through functions that allow recognition of the person’s blob and control of its geometric characteristics through anthropometric thresholds. For these reasons, it is possible to compare the relative height of the person’s blob with respect to the floor [10]. We retained the top view configuration instead of the front one, as it is more unobtrusive in a real scenario and more robust to possible occlusions due to objects located in the monitored environment (e.g., furniture). In this paper, different types of falls are analyzed, with the purpose ofing identify suitable functions for the automatic detection and classification of falls and non-falls obtained with high accuracy. In particular, we focused on the variation of the height of the person’s blob with respect to the floor to check if it is possible to discriminate falls, recovery situations, or activities. The classifier used in this approach is the support vector machine (SVM), and after defining some necessary parameters (e.g., the length of the sequence on which to calculate the features), the SVM training used by the algorithm is performed. The features that we called the depth values histograms (DVHs) are the histograms calculated on the distance values between the sensor and the person’s blob, extracted from the frame depth, normalized with respect to the floor distance. Once the features are acquired, the data are organized to proceed with the training and testing of the SVM. We trained the SVM with a linear kernel, and the SVM model was built with the leave one actor out technique [11] and trained with the DVH features obtained considering all the actors (from 1 to 20) for the training. The system accuracy is the leave one actor out test result.

The paper is organized as follows: Section 2 provides an overview of the related work from the literature about fall detection. Section 3 concerns the details of the proposed method, while Section 4 highlights the algorithm performance. Finally, Section 5 concludes the work and suggests further developments.

## 2. Related Works

The present paper took the basis and extended the previous method [10] in which an ad-hoc person identification method based on the Sobel edge detection algorithm [12], allowed the extraction of a useful blob relative to the person with the aim of identifying a fall when the person’s blob was sufficiently close to the floor height. Despite the effectiveness of the method, not all types of falls were recognized. In fact, when the centroid associated to the person remained above a threshold in height (e.g., when the person fell on their knees), the fall was not identified. Several algorithm problems have been solved by developing additional functionalities in the method presented herein. To solve the failures and optimize the functionalities of the previous study, we took inspiration from some literature described in this section.

Several approaches based on a single Kinect device mounted on the ceiling [13,14,15,16] revealed the usefulness and robustness of fall detection methods developed with this sensor. Amini et al. [16] used two fall detection approaches, heuristic and based on machine learning with the *AdaBoostTrigger* algorithm. Their work showed a 95.42% accuracy in the heuristic approach and 88.33% in the machine learning technique.

Kepski et al. [17], like the work here presented, also used a ceiling-mounted Kinect. For their study, they started the analysis of the depth images after the indications coming from a body-worn tri-axial accelerometer for a potential fall. They used the k-nearest neighbors (k-NN) classifier, trained on features relative to head–floor distance, person area, and human shape’s max length to width to distinguish a lying pose from other daily activities in the depth images with a low false positive rate. They used a great number of depth images with fall-like activities and simulated falls obtaining a low level of false alarms corresponding to an error of 0%.

In the same year, Feng et al. [18] used an ellipse to fit the human segmented silhouette and extracted several features correlated to the human body deformation by the integrated normalized motion energy image (INME). They classified four different human postures during the fall via a multi-class SVM and a context-free grammar-based method, obtaining the detection of falls with a sensitivity of 95.2%.

In a recent study [19] in which the Kinect sensor is used in a top-view configuration, the authors trained a binary SVM extracting features from an elliptical bounding box fitted to the human silhouette. The classifier showed an accuracy of 96.3% by testing over 10 top-viewed depth camera video images of the UR (http://fenix.univ.rzeszow.pl/mkepski/ds/uf.html) fall detection dataset [20] in which accelerometer data is also provided.

Aziz et al. [21] investigated the performance of a fall detection system based on real-world fall and non-fall data sets. They recorded 400 hours of ADLs from nine participants recruited through the RBK Geriatrics Department, who performed the tests in two months. They used a machine learning algorithm based on an SVM classifier to discriminate falls from non-fall events, and their developed system showed an extremely low false positive rate, with 80% sensitivity and 99.9%

Nizam et al. [22] discriminated falls from ADLs, extracting skeleton joint coordinates from depth data to consider the subjects’ height and velocity as training features for the classification process. The velocity showed differences between lying on the floor and falling. The change in the magnitude of the velocity vector was more remarkable for falls than for the lying on the floor activity. The authors of this work stated that their system was characterized by an accuracy of 93.75%, a sensitivity of 100%, and a specificity of 92.5%.

Accuracy could be increased by exploiting wearable devices. De Quadros et al. [23] used a wrist-worn fall detection solution and declared in their study to achieve an accuracy of 91.1%, using a threshold-based method for the acceleration data, and an accuracy of 99.0% with a machine learning approach, in particular the k-NN method.

Although the use of a wearable device may provide a good solution, like the authors declared, the aim of the study here presented is to obtain a high system accuracy offering an almost invisible and no-contact solution to the subjects moving in a room. In the present paper, an unobtrusive solution for fall detection and discrimination between falls and ADLs is proposed, taking into consideration 32 falls and 8 ADLs simulated by 20 volunteer subjects. A classifier based on binary and multi-class SVM is used, and respectively two-class and three-class systems have been considered to show the resulting system accuracy.

## 3. Method Details

The first phase of the proposed work was concerned with the definition of a protocol to perform different types of falls and ADLs, and therefore the collection of a dataset from the subjects participating in the tests.

### 3.1. Dataset and Protocol

**Figure 1:**
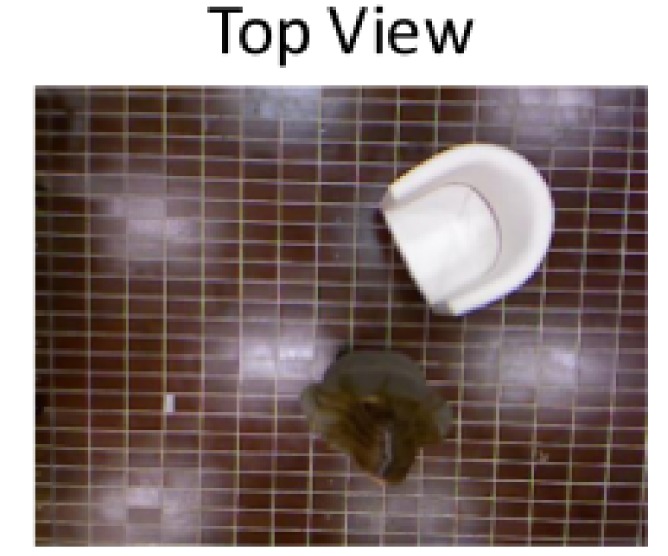
The setup consists of a sensor placed on the ceiling of the laboratory at a height of 3 m from the floor. The subject in the picture is performing the *ASSO* activity of daily living (ADL).

The definition of the dataset and protocol used for the ADLs and falls detection algorithm was obtained considering the sensor setup shown in Figure 1, which was installed on the ceiling in a top-view configuration, and the project requirements that can be summarized as follows:

- facility to detect falls in which the person ends lying on the ground;
- ability to detect falls in which the person finishes sitting on the ground;
- ability to detect falls in which the person falls on the ground finishing on the knees, possibly interacting with objects in the environment;
- ability to track the person’s recovery, and discriminate the fall in which the subject remains on the ground with respect to the one in which the person is able to get up (recovery).

Considering the previously described requirements and the sensor setup, the different types of falls and ADLs need to be included in a protocol. The ADLs were chosen as the typical situations of daily living that the subjects carry out at home and that could be confused with the falls of interest, generating false alarms. The data collection protocol consisted of the sequences shown in Table 1. In particular, the simulated falls can be divided into eight groups: backward finishing lying, backward finishing sitting, forward finishing lying, forward finishing on knees, forward finishing on knees grabbing a chair, forward finishing on knees grabbing a sofa, left side, and right side. Four different situations were considered for each subject that participated in the tests:

1. he/she falls from standing position and then remains on the ground;
2. he/she falls from standing position and then recovers;
3. he/she falls during walking and then remains on the ground;
4. he/she falls during walking and then recovers.

The performed activities can be divided into four groups: pick up an object from the floor with bending, pick up an object from the floor with squatting, sit and get up from a chair, sit and get up from a sofa. For each of them, two different situations have been considered:

1. at the beginning from walking and, after the task, walking again;
2. at the beginning from a standing position and, after the task, standing up again.

The protocol was performed by 20 healthy subjects, aged between 21 and 55 whose characteristics are shown in Table 2. The average age (±standard deviation) of the participants was 28 ± 8.7 years, with an average height of (176 ± 8.1) cm for males and (163 ± 4) cm for females, and average weights of (72 ± 8.4) kg for males and (61 ± 8.5) kg for females. Each subject performed all 32 fall actions and 8 actions related to ADLs, generating a total dataset of 800 sequences. In addition to the sequence of frames representing the action, for each acquisition a background sequence was also captured, which is relative to the scene setup without the presence of the person. The acquisitions were conducted with the Microsoft Kinect v1 sensor, storing the following information:

- depth: depth frame in 320 × 240 format, captured at 30 fps ;
- RGB: RGB frame in the 640 × 480 format, captured at 30 fps;

The RGB frames were captured only to acquire color information of the subjects by an image captured in the top-view configuration, but the RGB frames were not used in the processing of this work. The principal aim of the proposed work is to preserve subjects’ privacy, and for this reason we decided to use only the depth frames for data processing. Indeed, thanks to the depth frames, we were able to recognize the person’s blob like a point cloud and extract geometric features useful to discriminate falls and ADLs in a domestic environment, friendly to the subjects under test. The tests were conducted in our laboratory as a preliminary analysis with the aim of realizing in the future further tests in homes, hospitals, and health care facilities.

### 3.2. Parameters Setting and Algorithm Specifics

The proposed algorithm requires the definition and setting of different thresholds, resumed as follows. The following parameters were chosen empirically considering the amount of time after which a person could be considered in a fall condition. In this work, we estimated a time of few seconds, maximum 3 s, for recovery after a fall.

**Figure 2:**
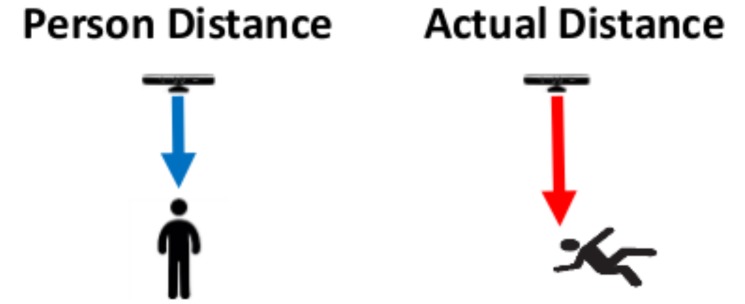
The person’s distance from the sensor is the distance when the person is standing. The actual distance, on the right, is the person’s distance from the sensor in the actual processed frame.

- *SensorHeight* = 3000 mm; height at which the Kinect sensor is installed with respect to the floor.
- *FloorDistance* = 600 mm; threshold height from the floor.
- *thresholdFall* = *SensorHeight* − *FloorDistance*; if the person’s distance from the sensor, in the current frame, exceeds this quantity defined *thresholdFall*, then the person is considered as near the floor, and therefore a fall or warning can be notified. Figure 2 shows the person’s distance from the sensor in the frame in which the person is standing, on the left, and in the current processed frame, on the right.
- *wind_time* = 3 s; time chosen to evaluate the presence of a fall or warning.
- *recovery_time* = 2 s; maximum time for which the subject can be detected on the ground without the identification of falls.
- *warning_time* = 1 s; maximum time for which the subject can be detected on the ground without warning notification.
- *sit_time* = 3 s; time duration of the sequence over which the features are extracted from the depth frames.
- *shift_time* = 1 s; portion of the buffer to be removed before extracting the features from the depth frames.
- *wait_time* = 1 s; waiting time after an ADL identification through SVM before restarting the evaluation.
- *features type* = *Depth Values Histogram, DVH*; type of features to be calculated from the depth data to use the SVM classifier. The DVH features are represented by the histograms calculated on the distance values of the depth frame normalized with respect to the distance from the floor.
- *numBins* = 256; number of bins to consider for the histograms computation in the DVH features.

**Figure 3:**
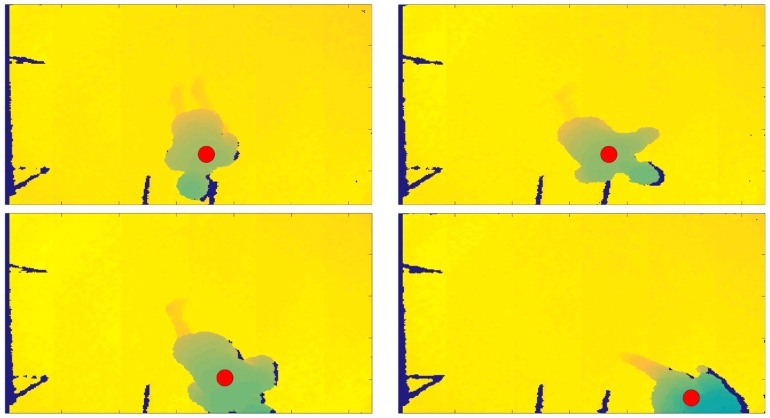
Recovery phase of the *FFOKCHSTRC* sequence simulated by *ES*01, where the person left the scene after a fall without the correct acquisition of the initial height. The dot is a marker that identifies a fall when it is red.

**Figure 4:**
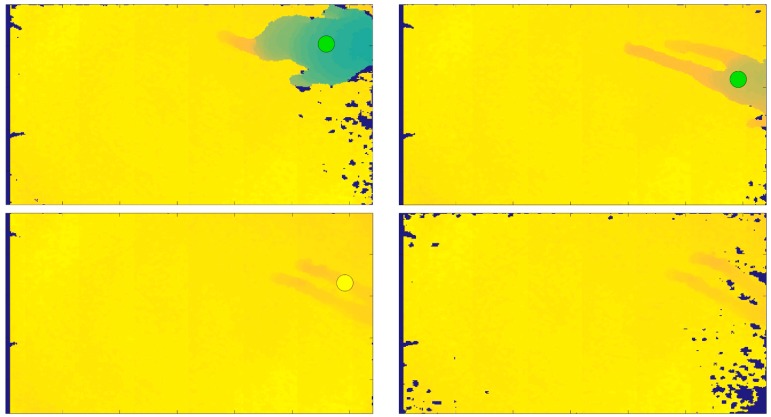
Fall phase of the FFELFRWK sequence simulated by ES09, where the tracking was lost because the person was out the sensor coverage area for most of their body parts.

**Figure 5:**
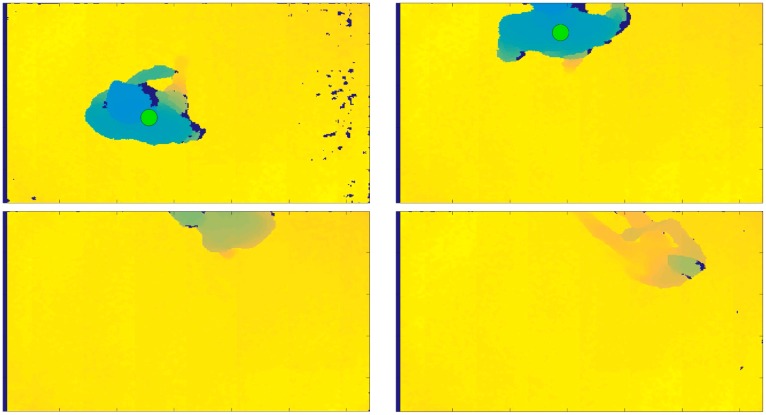
Fall phase of the *FBELFRWK* sequence simulated by *ES*14, where the person entered the sensor coverage area but then left the scene before falling.

The proposed fall detection algorithm was developed using two programming environments. To acquire the depth frames with Kinect v1 in real-time, we used the Software Development Kit (SDK) v.1.5., through Microsoft Visual Studio, starting with the available code Depth Basics-D2D C++ (https://msdn.microsoft.com/en-us/library/hh973081.aspx) and introducing the ad hoc functionalities for automatic person identification. A sample functionality introduced in the developed tool is the person’s identification by the sensor, represented by a dot that changes in color, as shown in Figure 3, Figure 4 and Figure 5. The dot is green when the person is standing below the sensor, is yellow when a warning condition occurs, and is red when the algorithm identifies a fall. The data were processed using Matlab programming environment, in particular to extract the classification process outcomes.

### 3.3. Fall Detection Algorithm

The developed fall detection algorithm took its basis from the system described in [10]. This system was able to detect and monitor a person in subsequent frames, and a fall was identified when the distance between the Kinect and the centroid associated with the person’s head became comparable with the floor distance. This means that when the person was on the ground but the centroid was not close to the floor, such as the case in which the person falls finishing on their knees, the fall was not detected. Considering the protocol described in Section 3.1, the aforementioned conditions could be verified in cases of falls on the knees (*FFOKxx*), or when the person fell finishing sitting on the ground (*FBESFR*) with the centroid placed on the shoulders or on the head.

**Figure 6:**
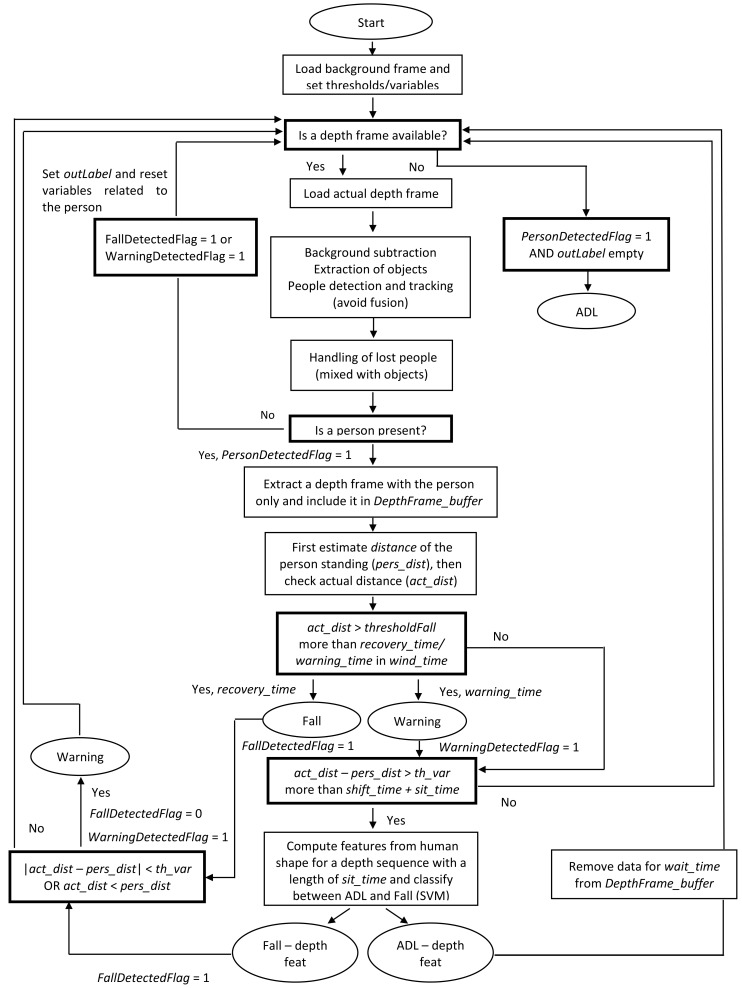
Main scheme of the proposed system.

The work proposed herein extends the functionality of the basic algorithm by defining a system able to manage all the situations included within the protocol. The flow diagram of the algorithm is described in Figure 6, showing the first operations concerning the background acquisition and the setting of the thresholds related to the parameters necessary for the functioning of the whole system. A depth frame is then processed to remove the background and discriminate the different objects in the scene from the person [10].

If the algorithm detects a person within the scene, a depth frame is extracted with the same resolution as the starting depth frame (320 × 240), but in which all the pixels that do not belong to the subject’s blob are eliminated and the foreground is highlighted. This frame, with only the foreground, is then inserted into a buffer (called *DepthFrame_bu f f er*) which contains the data related to the last *sit_time + shi f t_time* seconds, which are parameters to set as time windows. If the person is detected for the first time, an estimate of the distance between the sensor (installed on the ceiling) and the centroid located on the person’s head is calculated. This distance, defined as *pers_dist*, is stored in the first frames where the person is identified. The same distance in the current frame can be defined as *act_dist*, and is monitored to evaluate if the person is close to the floor, a situation recognized when the following condition is verified:

(1) act_dist>thresholdFall.

To detect a risky condition (i.e., a warning), a time window in seconds called *wind_time* is considered and the evaluation of the distance between the person and the floor is calculated with a double control. First, if the person is close to the floor for a time interval greater than *recovery_time*, a fall is identified by labeling the sequence as “Fall”; on the contrary, if the person is close to the floor for a time grater than *warning_time* seconds, a “Warning” condition is classified. We experimentally defined a threshold as 15% of the difference between the height at which the Kinect sensor is installed to the ceiling, called *SensorHeight*, and the *pers_dist*:

(2) th_var=0.15(SensorHeight−pers_dist).

The system at this point is subjected to a second control; in fact, it continues to monitor the current distance of the person and to compare this distance with respect to the corresponding *pers_dist* to detect a possible recovery phase. In this case, the expression (3) is validated and the condition previously labeled with *Fall* is changed to *Warning*.

(3) ∣act_dist−pers_dist∣<th_varoract_dist<pers_dist

When a warning condition occurs, the expression

(4) act_dist−pers_dist>th_var

is checked. If the expression (4) is validated for a time window greater than the sum between *shi f t_time* and *sit_time* seconds, the *DepthFrame_bu f f er* is considered only for the frames relative to the last *sit_time* seconds and the features of the person’s blob from the depth frames are calculated.

The next step consists of the analysis of the person tracking to check if the person that was present in the previous frame was labeled as an object in the current frame. This check is processed only if the person is no longer identified but more objects are detected in the current frame than in the previous frame, which is the typical situation of fusion between a person and objects. If these conditions are verified, the computation of the features from the human shape is processed by an SVM binary algorithm, that we called the depth value histogram (DVH). The calculation of the DVH features consists of the comparison of the blob relative to the person in the previous frame and the blobs related to the neighboring objects in the current frame. In this case, the histograms are compared and the association occurs if the sum of the differences between the histogram bins is lower than a specific threshold. In particular, three processing steps are required to extract features from depth frames:

- construction of a matrix that contains a sequence of depth frames from the sensor that has to be processed (*row pixels* × *column pixels* × *number of f rames*);
- creation of a structure relative to the feature type computed on depth data output;
- creation of a matrix with features computed from the sequence of data (*number of features* × *number of frames*).

For each depth frame, an array of features is calculated and processed by the SVM, an algorithm able to discriminate each activity between falls and ADLs, classifying them with the correct label. Since the SVM, for each depth sequence, is processed for a time window equal to *sit_time* × *fps*, where fps stands for frames per second, the final outcome of the classification process is decided by a majority criterion: if more than half of the frames were associated with a fall, the sequence is labeled as *Fall_depth feat*. On the contrary, if the majority of activities is identified, the sequence is recognized as *ADL-depth feat*. *recovery_time* (continuously), a fall is detected considering the depth features and a flag contained into an array of flags related to the detected fall is set to one. If the the array of flags contains more “ones” than “zeros”, the *Fall_depth feat* is labeled, otherwise an *ADL-depth feat* is labeled. An ad hoc developed function allowed the calculated features to be acquired and organize data to proceed with SVM training and testing. The script hence manages the balancing of the dataset (since there are more sequences of fall than sequences of ADL) and the normalization of data. With this function, a sparse solution of SVM for decision hyperplane is used. In particular, to have a comparable amount of data for the different classes, replicas of the acquired depth images were created by rotating them.

If the falls are classified through the SVM, the system continues to monitor the person’s distance from the floor. If the SVM detects the presence of an ADL, a mechanism was introduced to prevent control repetitions at each new frame. This mechanism consists of removing part of the *DepthFrame_bu f f er*, eliminating a number of frames corresponding to *wait_time* seconds. The sequence is labeled as ADL, when all the frames were processed, if a person was identified, but none of the previously stated conditions were not validated. In particular, the previous conditions are related to the person’s height with respect to the floor and the sensor’s height compared according to the expressions and mathematical inequalities (1)–(4). The ADLs performed are characterized by actions in which the person is never close to the ground for a time greater than some time interval, described in Section 3.2, chosen to discriminate between falls and activities.

## 4. Results and System Performance

**Figure 7:**
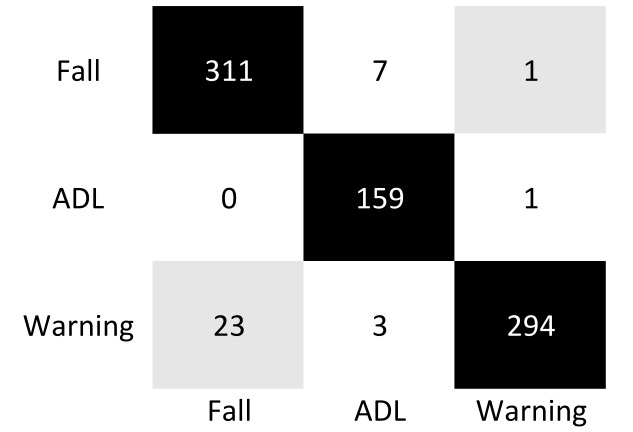
Confusion matrix resulting from the fall detection system.

Figure 7 shows the confusion matrix resulting from the training test carried on the complete dataset. Considering the Warning events as a distinct dataset class, we could identify 3 classes and overall 800 sequences of which 160 were ADLs, 320 Fall, and 320 Warning. With this multi-class approach and a one-actor-vs.-rest method, the resulting accuracy was 95.6%, taking into account that the system did not classify a single dataset sequence because the person was not recognized (799 sequences instead that 800 were recognized by the system). Assuming instead to consider a classification process with two outcomes as system output, considering Fall and Warning as a single class, the system performances increased as shown in Table 3.

As shown in Figure 7, the most frequent problem was the recognition as Fall of a sequence labeled as Warning (23 occurrences in the confusion matrix shown in the Figure 7). The classification system monitors the person constantly, taking into account the height before and after a Fall, Warning, or ADL event. However sometimes, the recovery after a fall performed by a subject, event that should be classified as a Warning condition, was recognized as a Fall.

Another problem occurred when the person was not identified by the sensor, because entering the coverage area of the sensor without the previous correct acquisition of their height (in fact we had TP = 629, as shown in Table 3). This problem is clearly visible in Figure 3, where during a fall, in particular the *FFOKCHSTRC* sequence performed by *ES*01, the centroid associated to the person was never positioned on the head. This problem occurred because the person entered in the scene falling in the first frame acquired by the sensor, too fast with respect to the time that the algorithm needs to recognize the person centroid on the head.

Another situation that occurred which led to the incorrect classification of fall sequences is the fall FFOKSOWK performed by subject ES01. In this case, the sequence was incorrectly recognized as an ADL because the person’s recovery happened too quickly and the time windows, set as a time limit in order for an action to be identified as a Warning condition, was not respected. Indeed, in this case the person’s height variation did not respect the time condition to be classified as warning, which is more than *shi f t_time + sit_time* seconds, as indicated in expression (4). Since the sum of these two temporal windows was empirically set to 4 seconds, it is possible to assume that this is a problem related to some sequences of the dataset and that, usually, in the real context, a generic fall implies to remain on the ground for at least 4 seconds before proceeding to a possible recovery. To define temporal windows, we considered that, in accord with Montanini et al. [24], a too-narrow temporal window would cause more false positives, while a too-large one would make the classification system less accurate, especially in the case of recoveries. Alternatively, these temporal windows could be reduced and even shorter sequences could be analyzed.

**Figure 8:**
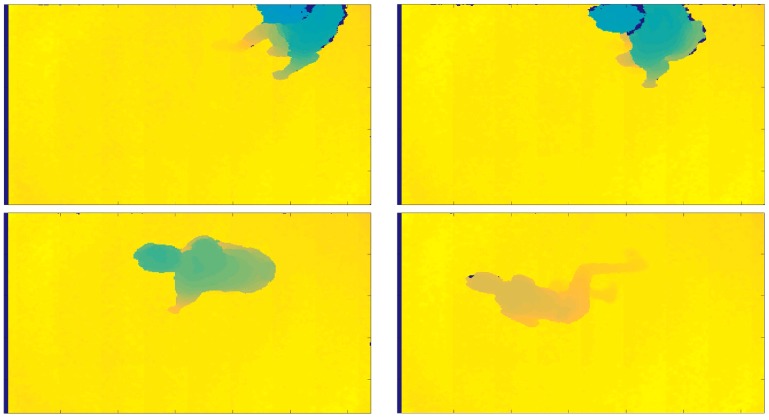
Fall phase of the *FSRIFRWK* sequence simulated by *ES*11, where the person was not recognized. The dot is not present in the figure.

Some SVM classification errors occurred in the case of *ASCHWK* performed by subject *ES*04, in which an ADL was recognized as a Fall and *FFOKFRST*-*STRC* simulated by *ES*08, in which a Fall was recognized as an ADL. In one single case (*FSRIFRWK* performed by *ES*11) the person was not recognized, and this was caused by the unusual position in which the person entered the scene, as the sequence shows in Figure 8.

Another critical situation occurred when the subject fell with the body almost entirely outside the sensor coverage area, as shown in Figure 4. In this sequence (*FFELFRWK* performed by *ES*09), the person tracking with the blob was lost when the person stood on the ground and the action was labeled as an ADL because the person was not identified and the correct fall condition was not satisfied.

Another undetected fall situation was *FBELFRWK* simulated by *ES*14, in which the person entered the scene and was correctly recognized, but then left the field of view before falling to the ground, as shown in Figure 5.

In all other sequences, the system detected and correctly classified each type of fall or activity performed by the subjects. The outcomes in Table 3 allowed us to calculate the two-class system accuracy (Ac).

The Ac of the three-class system, with the warning cases as a separate class of the dataset, is calculated as in the following expression:

(5) $$Ac_{\left(3-classes\right)}=\frac{\mathrm{total}\mathrm{correct}\mathrm{predictions}}{\mathrm{total}\mathrm{predictions}\mathrm{made}}\times 100=95.6\%$$

The Ac of the two-outcome system, calculated considering falls and warning conditions in the same class, is visible in expression (6). The Ac was obtained considering that TP = 629, TN = 159, FP = 1, FN = 10.

(6) $$Ac_{\left(2-classes\right)}=\frac{TP+TN}{TP+FP+TN+FN}=98.6\%$$

The Ac is the correct classification rate [25], and it was calculated considering the SVM models trained with the obtained features, setting DVH as the classification feature type with 256 bins.

## 5. Conclusions and Future Works

The method developed in the proposed work consisted of three main blocks: real-time acquisition of the height of the maximum point associated to the person and extraction of the blob area of the person from the depth frame; features extraction starting from the depth data; features processing and SVM models definition. The organization in three steps allowed us to perform multiple tests, with different parameters, for each step independently. The system accuracy showed that the proposed method is a valid approach to identify and classify the different types of falls and ADLs. In particular, the accuracy of the two-outcome system, equal to 98.6%, is greater than the system accuracy obtained from the works discussed in Section 2 [16,19,22], which were taken as references for the study presented herein. However, system errors occurred for the most part due to the non-regular entry of the person into the sensor coverage area. A possible future solution could be the installation of multiple Kinect sensors [26] to cover a greater area than the actual of 8.25 m^2^, so as to increase system accuracy and reduce the noise present in the border as shown in Figure 4 and Figure 5a. The noise can also be removed from the depth frames by applying post-processing filtering techniques [27]. The noise in the depth frame boundaries could often lead to non-detection of a subject that performs a fall in the edge area and not in the center of the scene acquired by the sensor.

## Figures and Tables

**Table 1:** Protocol of fall detection experiments.

Type	Prefix Name	Suffix Name	Description
Backward fall, finishing lying	FBELFR	ST	Subject is standing, falls backwards, and remains on the ground.
	FBELFR	STRC	Subject is standing, falls backwards, stays on the ground
			for a while, and then gets up again.
	FBELFR	WK	Subject walks, falls backward, and remains on the ground.
	FBELFR	WKRC	Subject walks, falls backward, stays on the ground
			for a while, and then gets up again.
Backward fall, finishing sitting	FBESFR	ST	Subject is standing, falls backwards, and remains on the ground.
	FBESFR	STRC	Subject is standing, falls backwards, stays on the ground
			for a while, and then gets up again.
	FBESFR	WK	Subject walks, falls backward, and remains on the ground.
	FBESFR	WKRC	Subject walks, falls backward, stays on the ground
			for a while, and then gets up again.
Forward fall, finishing lying	FFELFR	ST	Subject is standing, falls forwards, and remains on the ground.
	FFELFR	STRC	Subject is standing, falls forwards, stays on the ground
			for a while, and then gets up again.
	FFELFR	WK	Subject walks, falls forwards, and remains on the ground.
	FFELFR	WKRC	Subject walks, falls forwards, stays on the ground
			for a while, and then gets up again
Forward fall on the knees grabbing the chair	FFOKCH	ST	Subject is standing, falls forwards, and remains on the ground,
			grabbing the chair.
	FFOKCH	STRC	Subject is standing, falls forwards, stays on the ground
			grabbing the chair for a while, and then gets up again.
	FFOKCH	WK	Subject walks, falls backward, and remains on the ground,
			grabbing the chair.
	FFOKCH	WKRC	Subject walks, falls backward, stays on the ground
			grabbing the chair for a while, and then gets up again.
Forward fall on the knees	FFOKFR	ST	Subject is standing, falls forwards, and remains on the ground.
	FFOKFR	STRC	Subject is standing, falls forwards, stays on the ground
			for a while, and then gets up again.
	FFOKFR	WK	Subject walks, falls forwards, and remains on the ground.
	FFOKFR	WKRC	Subject walks, falls forwards, stays on the ground
			for a while, and then gets up again.
Forward fall on the knees grabbing the sofa	FFOKSO	ST	Subject is standing, falls forwards, and remains on the ground,
			grabbing the sofa.
	FFOKSO	STRC	Subject is standing, falls forwards, stays on the ground
			grabbing the sofa for a while, and then gets up again.
	FFOKSO	WK	Subject walks, falls forwards, and remains on the ground,
			grabbing the sofa.
	FFOKSO	WKRC	Subject walks, falls forwards, and stays on ground
			grabbing the sofa for a while, and then gets up again.
Left side fall	FSLEFR	ST	Subject is standing, falls on their left side, and remains on the ground.
	FSLEFR	STRC	Subject is standing, falls on their left side, stays on the ground
			for a while, and then gets up again.
	FSLEFR	WK	Subject walks, falls on their left side, and remains on the ground.
	FSLEFR	WKRC	Subject walks, falls on their left side, stays on the ground
			for a while, and then gets up again.
Right side fall	FSRIFR	ST	Subject is standing, falls on their right side, and remains on the ground.
	FSRIFR	STRC	Subject is standing, falls on their right side, stays on the ground
			for a while, and then gets up again.
	FSRIFR	WK	Subject walks, falls on their right side, and remains on the ground.
	FSRIFR	WKRC	Subject walks, falls on their right side, stays on the ground
			for a while, and then gets up again.
Pick up object from floor with bending	APBE	ST	Subject is standing, bends, picks up an object on the floor, and then stands up again.
		
	APBE	WK	Subject walks, bends, picks up an object on the floor, and then stands up again.
		
Pick up object from floor with squatting	APSQ	ST	Subject is standing, squats, picks up an object on the floor, and then stands up again.
		
	APSQ	WK	Subject walks, squats, picks up an object on the floor, and then stands up again.
		
Sit and get up from the chair	ASCH	ST	Subject is standing, sits on a chair, and then stands up again.
	ASCH	WK	Subject walks, sits on a chair, and then stands up again.
Sit and get up from the couch	ASSO	ST	Subject is standing, sits on a couch, and then stands up again.
	ASSO	WK	Subject walks, sits on a couch, and then stands up again.

**Table 2:** Dataset of the subjects participating in the tests.

Subject	Sex	Age	Weight (kg)	Height (cm)
ES01	M	28	65	177
ES02	F	40	60	163
ES03	F	35	63	161
ES04	F	29	74	170
ES05	F	25	52	160
ES06	F	26	55	165
ES07	M	30	65	176
ES08	M	55	80	173
ES09	M	21	58	169
ES10	M	21	70	178
ES11	M	23	59	175
ES12	M	28	74	178
ES13	M	28	76	160
ES14	M	26	73	182
ES15	M	40	87	187
ES16	M	21	80	189
ES17	M	22	64	167
ES18	M	22	72	170
ES19	M	21	78	188
ES20	M	21	78	177

**Table 3:** Two-output system performances. Falls and Warnings are grouped in the same class and ADLs constitute a class apart.

Performances	Results
True Positives (TPs)	629
True Negatives (TNs)	159
False Positives (FPs)	1
False Negatives (FNs)	10

## Abbreviations

The following abbreviations are used in this manuscript:

AAL	Ambient Assisted Living
ADL	Activity of Daily Living
k-NN	K-Nearest Neighbors
INME	Integrated Normalized Motion Energy
SVM	Support Vector Machine
RGB-D	Red, Green, Blue-Depth
